# Transcriptome and Metabolome Analysis Provides Insights into the Heterosis of Yield and Quality Traits in Two Hybrid Rice Varieties (*Oryza sativa* L.)

**DOI:** 10.3390/ijms232112934

**Published:** 2022-10-26

**Authors:** Dahu Zhou, Xinyi Zhou, Changhui Sun, Guoping Tang, Lin Liu, Le Chen, Haohua He, Qiangqiang Xiong

**Affiliations:** 1 Key Laboratory of Crop Physiology, Ecology and Genetic Breeding, Ministry of Education, College of Agronomy, Jiangxi Agricultural University, Nanchang 330045, China; 2 Jiangsu Key Laboratory of Crop Genetics and Physiology/Jiangsu Key Laboratory of Crop Cultivation and Physiology, Agricultural College of Yangzhou University, Yangzhou 225009, China; 3 Jiangxi Academy of Agricultural Sciences Rice Research Institute, Nanchang 330200, China

**Keywords:** heterosis, transcriptome, metabolome, hybrid rice, WGCNA

## Abstract

Heterosis is a common biological phenomenon that is useful for breeding superior lines. Using heterosis to increase the yield and quality of crops is one of the main achievements of modern agricultural science. In this study, we analysed the transcriptome and metabolome of two three-line hybrid rice varieties, Taiyou 871 (TY871), and Taiyou 398 (TY398) and the parental grain endosperm using RNA-seq (three biological repeats per variety) and untargeted metabolomic (six biological repeats per variety) methods. TY871 and TY398 showed specific heterosis in yield and quality. Transcriptome analysis of the hybrids revealed 638 to 4059 differentially expressed genes in the grain when compared to the parents. Metabolome analysis of the hybrids revealed 657 to 3714 differential grain metabolites when compared to the parents. The honeydew1 and grey60 module core genes *Os04g0350700* and *Os05g0154700* are involved in the regulation of awn development, grain size, and grain number, as well as the regulation of grain length and plant height, respectively. Rice grain length may be an important indicator for improving the quality of three-line hybrid rice. In addition, the rice quality-related metabolite NEG_M341T662 was highly connected to the module core genes *Os06g0254300* and *Os03g0168100*. The functions of *Os06g0254300* and *Os03g0168100* are EF-hand calcium binding protein and late embroideries absolute protein repeat containing protein, respectively. These genes may play a role in the formation of rice quality. We constructed a gene and metabolite coexpression network, which provides a scientific basis for the utilization of heterosis in producing high-yield and high-quality hybrid rice.

## 1. Introduction

Heterosis refers to the phenomenon in which hybrid progeny have attributes that are superior to each of the parental strains in terms of yield, quality, and stress resistance [1,2]. Using heterosis is an effective method for improving rice yield and quality. Heterosis is primarily derived from allelic interactions between the parental genomes and is altered by genetic programming that promotes hybrid growth and fitness [3,4]. The yield traits of rice are controlled by quantitative biological processes with an extremely complex genetic mechanism [4]. The grain filling stage is fundamental to the formation of yield and quality and is of great significance for the study of heterosis [5,6]. Rice grain filling usually refers to the process of transporting photosynthetic compounds to the grain, which affects rice yield and rice quality [7]. We conducted a multiyear and multisite analysis of the yield advantage traits of multiple hybrid rice combinations and found that the number of florets per panicle and the number of effective panicles were the central factors responsible for the yield advantage of two-line hybrid rice [8]. The former has a superparental advantage, while the latter has a superpaternal advantage [8]. Thus, in the indica rice hybrid combination, the rice quality is better than that of the male parent because the grain shape of the hybrid is longer, the grain width is narrower, and the aspect ratio is increased, which is beneficial to the improvement in rice quality [9].

With the continuous development of modern molecular biotechnology and high-throughput sequencing technology, the use of genome-wide, transcriptomic, and metabolomic methods for analyzing the biochemical mechanisms underlying crop heterosis has become an increasingly important research direction [4,10,11]. The transcriptome refers to the collection of all transcripts in a cell at a specific developmental stage and under specific conditions [12]. Differences in gene expression are an important source of phenotypic diversity and the specific traits of diploid hybrids are the result of the coexpression of alleles from the male and female parents [13,14]. Compared with their parents, hybrids may have different allelic expression levels at specific loci, opening new directions for heterosis research [15]. The transcriptome and metabolome have been widely used in rice grain development and rice quality research [16,17]. Metabolites in rice can broadly reflect the overall metabolic state of rice seeds [18]. Metabolites in grains are the substances produced by rice that are most directly related to the ability to adapt to the environment and have a critical impact on rice quality [19].

In this study, three parents, Taifeng B, Guanghui 398, and Changhui 871, and two F1 hybrids, Taiyou 398 and Taiyou 871, were used as experimental materials. Transcriptome and metabolomic techniques were used to analyze the metabolic and genetic mechanisms related to yield and quality heterosis in three-line hybrid rice. These technologies and methods allow us to investigate the physiological and molecular mechanisms underlying the formation of yield and quality in the F1 generation of hybrids and to provide reference materials for the breeding of new high-quality and high-yielding rice varieties.

## 2. Results

### 2.1. Yield and Quality Analysis

In terms of yield, the YPP of TY871 was significantly higher than that of TF and CH (Table 1). The YPP of TY871 was 24.89% higher than that of CH. The YPP of TY398 was significantly higher than that of TF and GH. Thus, TY871 and TY398 showed significant heterosis in terms of yield. In terms of yield composition, the PL of TY398 was 13% and 17.51% higher than that of GH and TF, respectively, and the difference was significant. The PL of TY871 was 0.51% and 25.54% higher than that of CH and TF, respectively. The PBN and SBN of TY398 were significantly higher than those of TF and GH. The SBN of TY871 was 2.39% and 124.89% higher than those of CH and TF, respectively. The TGNPP of TY398 was 20.51% and 79.12% higher than that of GH and TF, respectively, and the difference was significant. The TGNPP of TY871 was 15.17% lower than that of CH, and the difference was significant. The SSR of TY398 was 1.63% and 15.77% higher than that of GH and TF, respectively. Among TY871, CH, and TF, TY871 had the highest SSR, and the differences was significant. The 1000-GW was higher in TY871 and TY398 and was significantly different from that in the other varieties. In conclusion, TY871 and TY398 showed strong heterosis in terms of yield.

The BR of TY871 was 0.43% and 2.05% higher than that of TF and CH, respectively, and there was a significant difference between TY871 and CH (Table 2). TY398 was significantly lower than GH. The MR of TY871 was 1.78% and 5.61% higher than that of TF and CH, respectively. The HMR of TY398 was lower than that of GH and TF to varying degrees. The CD was the lowest in TF, followed by TY871 and TY398. The degree of chalkiness of the male parent was higher than that among the two hybrid rice varieties. The GC of TF was the highest among the five varieties, followed by TY871 and TY398. The GC of TY398 was 15.27% higher than that of GH, and the difference was significant. The GC of TY871 was 31.29% higher than that of CH, and the difference was significant. The AC was the lowest in TF. The AC of TY398 was 6.53% lower than that of GH, although there was no statistically significant difference. The AC of TY871 was 6.72% lower than that of CH, and there was no significant difference. From the comprehensive data on rice quality, the quality of rice from the female sterile line TF was the best, and the quality of the rice from the male parent restorer lines GH and CH was poor. TY871 and TY398 integrated the rice quality traits of their male and female parents and showed heterosis.

### 2.2. Metabolite Profiles of Rice Grains

**Figure 1:**
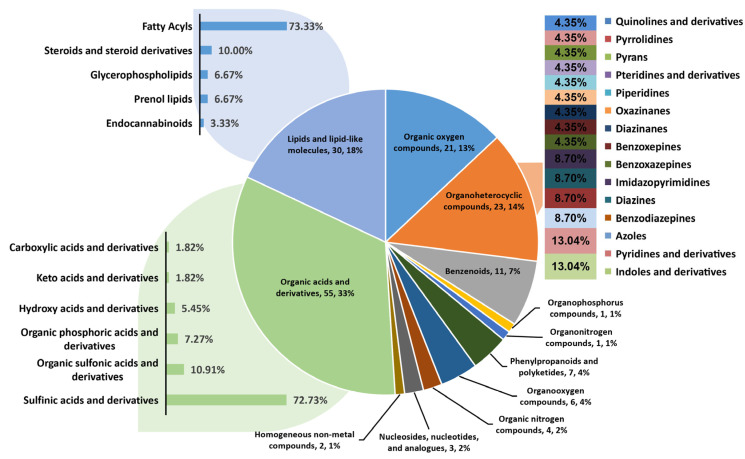
Classification and distribution of five sets of identified metabolites. The distribution of identified metabolites is shown in the pie chart, and subclassifications of amino acids, carbohydrates, and lipids are shown with bar charts and histograms.

To obtain an overview of the metabolic changes in the grains of the various rice tested varieties at the filling stage, nontargeted metabolic analysis was conducted with LC‒ESI‒MS/MS. In differential metabolite (DM) analysis, the metabolome samples were divided into five sets: CH-vs.-TY871, TF-vs.-TY398, TF-vs.-TY871, GH-vs.-TY398, and TY398-vs.-TY871. A total of 1455 DMs and 1304 DMs were identified in rice grains between CH and TY871 (Table S1) for metabolites enriched in positive and negative ion modes. A total of 2001 DMs and 1713 DMs were identified in rice grains between TF and TY398 (Table S2). A total of 1494 DMs and 1309 DMs were identified in rice grains between TF and TY871 (Table S3). A total of 2032 DMs and 1658 DMs were identified in rice grains between GH and TY398 (Table S4). Finally, 329 DMs and 328 DMs were identified in rice grains between TY398 and TY871 (Table S5). These metabolites were assigned to twelve superpathways (Figure 1) and 44 subpathways (Table S6) according to the Human Metabolome Database (https://hmdb.ca/, accessed on 18 July 2021). As expected, there was large variation in metabolite composition among the different rice varieties. Furthermore, clustering analysis was conducted for five sets of metabolites (Figure S1). Heatmaps of metabolites show similar changes for most compounds in TY398 and TY871. The map also shows the generally good biological reproducibility within the groups.

### 2.3. Transcriptome Profiles of Rice Grains

**Figure 2:**
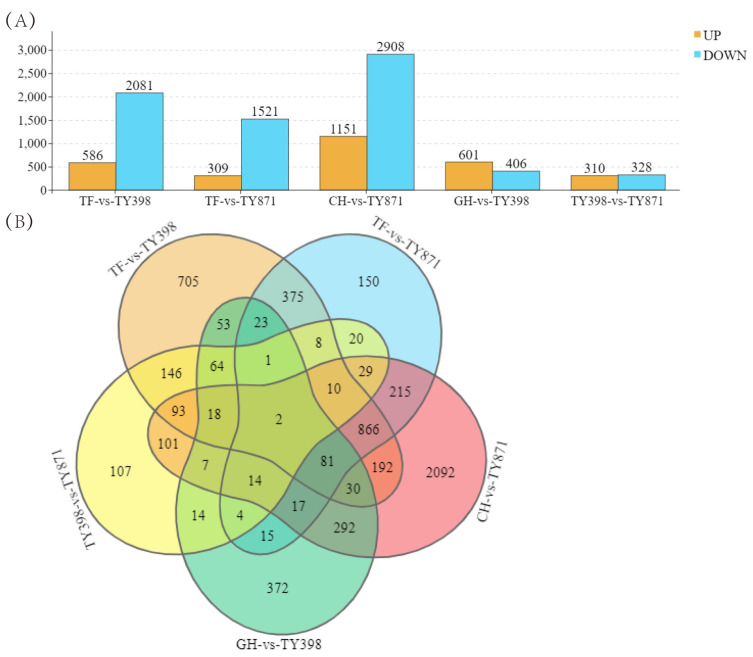
Differentially expressed genes analysis. (**A**) the number of differentially expressed genes in five sets (CH-vs.-TY871, TF-vs.-TY398, TF-vs.-TY871, GH-vs.-TY398, TY398-vs.-TY871); (**B**) Venn diagrams of the DEGs.

In DEG analysis, the RNA-seq samples were divided into five sets: CH-vs.-TY871, TF-vs.-TY398, TF-vs.-TY871, GH-vs.-TY398, and TY398-vs.-TY871. Among the 4059 DEGs, between CH and TY871 1151 were upregulated and 2908 were downregulated (Table S7; Figure 2B). Among the 2667 DEGs between TF and TY398, 586 were upregulated and 2081 were downregulated (Table S8; Figure 2B). Among the 1830 DEGs between TF and TY871, 309 were upregulated and 1521 were downregulated (Table S9; Figure 2B). Among the 1007 DEGs between GH and TY398, 601 were upregulated and 406 were downregulated (Table S10; Figure 2B). Among the 638 DEGs between TY398 and TY871, 310 were upregulated and 328 were downregulated (Table S11; Figure 2B). The DEGs of the five sets are shown in Venn diagrams in Figure 2C.

To further understand the functions of the DEGs and the related biological processes in which they participate, GO and KEGG enrichment analyses were conducted. GO analysis classified DEGs in the five sets (CH-vs.-TY871, TF-vs.-TY398, TF-vs.-TY871, GH-vs.-TY398, and TY398-vs.-TY871) into molecular function, cellular component, and biological process groups, involving 49 GO terms (Figure S2A). Within the molecular function category, the enriched GO terms were mainly catalytic activity and binding. Within the cellular component category, the enriched GO terms were mainly cells. Within the biological process category, the enriched GO terms were mainly metabolic process and biological regulation. Pathway enrichment analysis of the DEGs identified in the present study using KEGG showed that carbohydrate metabolism, amino acid metabolism, metabolism of cofactors and vitamins, biosynthesis of other secondary metabolites, energy metabolism, and lipid metabolism were significantly enriched (Figure S2B). The transcription and metabolism analysis results consistently showed that rice grain quality formation during grain filling significantly affected amino acid metabolism, energy metabolism, and carbohydrate metabolism.

### 2.4. Identification of WGCNA Modules Associated with Yield and Rice Quality in Different Varieties

**Figure 3:**
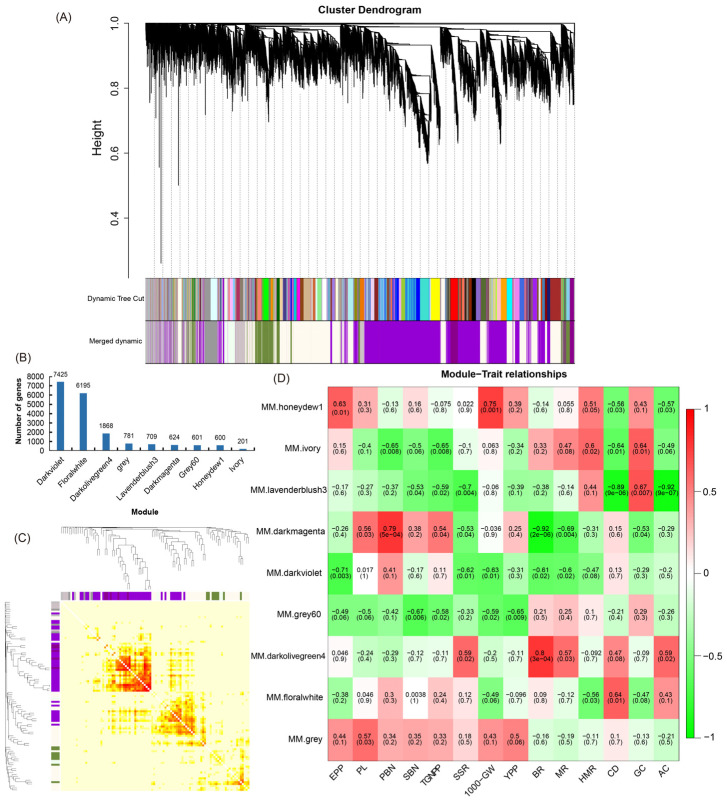
Gene module analysis. (**A**) module hierarchical clustering diagram. Dynamic Tree Cut was used to divide modules according to clustering results. Merged Dynamics was used for module division after merging the modules with similar expression patterns according to the module similarity, and the subsequent analysis was carried out according to the merged modules; (**B**) histogram of the number of genes in each module. The horizontal axis represents each module, and the vertical axis represents the number of genes; (**C**) modular gene correlation heatmap. Each row and column represents a gene, and the darker the color of each point (white→yellow→red) represents stronger connectivity between the two genes corresponding to the row and column, that is, the stronger the Pearson correlation. (**D**) correlation graph of trait association. The abscissa is the trait, and the ordinate is the module. Red represents a positive correlation, and green represents a negative correlation; the darker the color is, the stronger the correlation. The number in the brackets represents a significant *p*-value, where the smaller the value is, the stronger the significance.

To make the coexpression network conform to the scale-free network distribution, the optimal soft threshold β = 18 was determined (Figure S3A), and the network connectivity under different soft thresholds was shown, which was further used to construct the coexpression network (Figure S3B). WGCNA was performed on 19,004 genes (Figure 3A,B). A gene module refers to a collection of highly related genes, and genes in the same module may have similar biological functions. We analyzed nine of these modules, of which the dark violet module had the largest number of genes, with 7425; the ivory module had the smallest number of genes, with 201 (Figure 3B). Correlation analysis was performed on the expression levels of each gene and the module eigenvalues (Figure 3C), and the expression patterns of the module genes in each sample were displayed by the module eigenvalues. The module eigenvalue is equivalent to the weighted composite value of all gene expression values in the module. Through the sample expression pattern heatmap, we determined which modules were significantly related to specific samples so that the corresponding modules could be selected for further research. Analysis of the module–trait relationships for 18 samples revealed that EPP (R = 0.63 and *p* = 0.01) and 1000-GW (R = 0.75 and *p* = 0.001) were significantly positively correlated with the honeydew1 module (Figure 3D). TGNPP (R = −0.65 and *p* = 0.008) and PBN (R = −0.65 and *p* = 0.008) were significantly negatively correlated with the ivory module (Figure 3D). YPP (R = −0.67 and *p* = 0.006) and SBN (R = −0.65 and *p* = 0.009) were significantly negatively correlated with the grey60 module (Figure 3D). CD (R = −0.89 and *p* = 9 × 10^−6^) and AC (R = −0.92 and *p* = 9 × 10^−7^) were significantly negatively correlated with the lavenderblush3 module (Figure 3D). GC (R = 0.67 and *p* = 0.007) was significantly positively correlated with the lavenderblush3 module (Figure 3D). Therefore, honeydew1, ivory, grey60, and lavenderblush3 modules can be used as target gene modules. The expression levels of all genes and eigenvector genes in these four modules were analyzed separately in all samples, and it was found that the gene expression levels within each module were highly correlated. The expression level of eigenvector genes was also highly correlated with the overall expression level of the module, indicating that the eigenvector genes of a target module can adequately represent the overall genes in its module (Figure S4).

### 2.5. Functional Annotation of Hub Genes Highly Associated with Yield and Quality Traits

**Figure 4:**
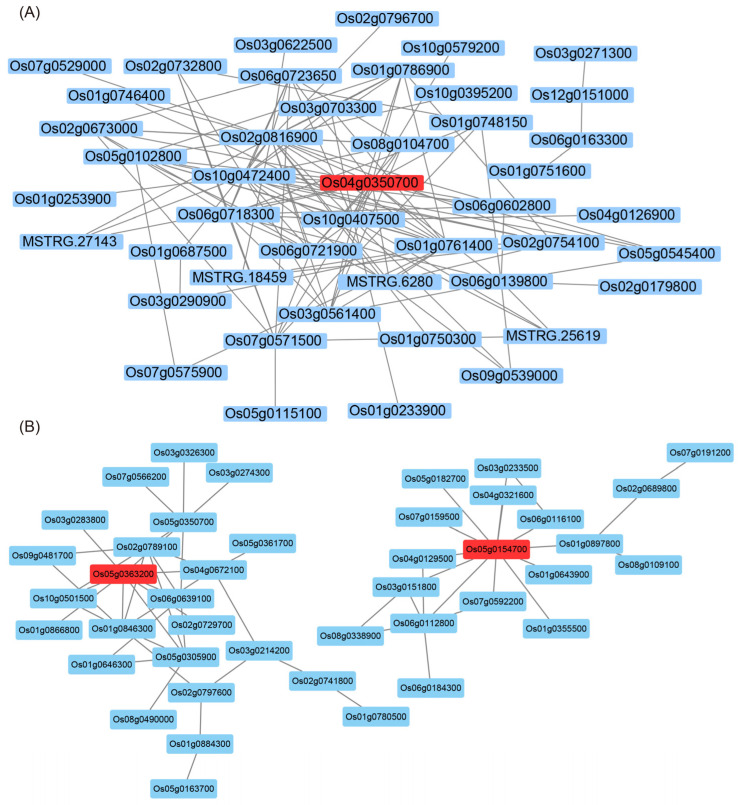
Gene coexpression networks within the honeydew1 (**A**) and grey60 (**B**) modules and their core genes.

**Figure 5:**
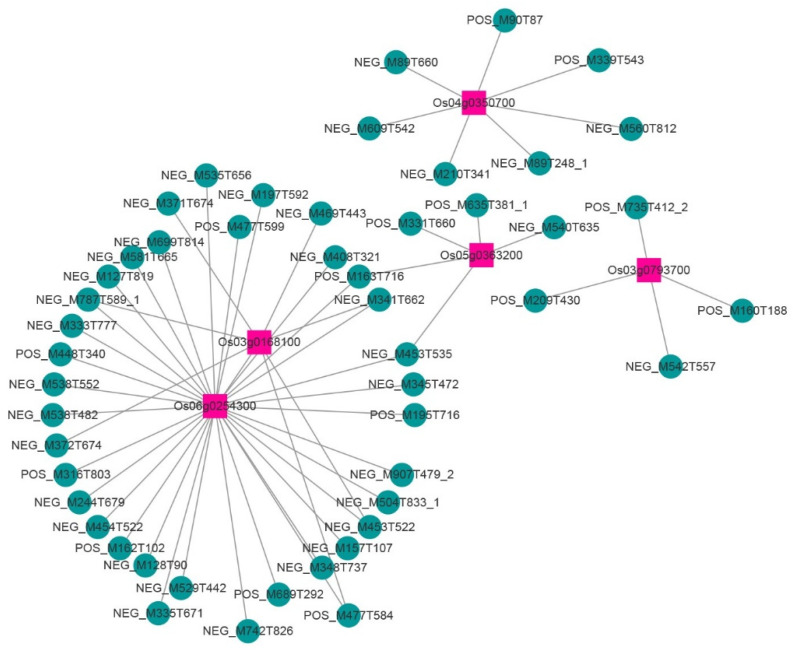
Core gene and metabolite connectivity network diagram.

**Figure 6:**
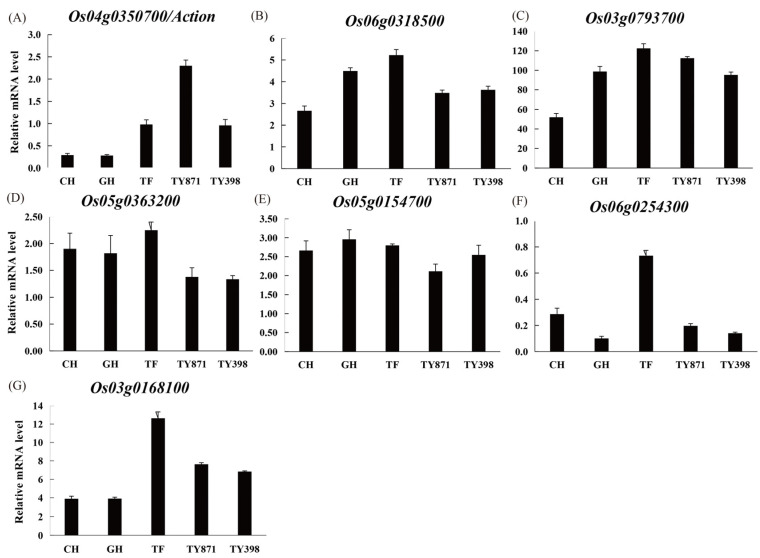
qRT‒PCR validation of the seven genes from RNA-seq analysis. (**A**) Relative mRNA expression level of *Os04g0350700* gene. (**B**) Relative mRNA expression level of *Os06g0318500* gene. (**C**) Relative mRNA expression level of *Os03g0793700* gene. (**D**) Relative mRNA expression level of *Os05g0363200* gene. (**E**) Relative mRNA expression level of *Os05g0154700* gene. (**F**) Relative mRNA expression level of *Os06g0254300* gene. (**G**) Relative mRNA expression level of *Os03g0168100* gene.

To obtain the core genes in the above four modules, Cytoscape software was used to visualize the gene regulatory network, and the genes with high connectivity in each module were identified and used as the core genes in each respective module (Figure 4; Figure S5). One core gene was found in the top 125 genes of the honeydew1 module (Figure 4A). The top 80 genes in the ivory module revealed two core genes (Figure S5A). The top 89 genes in the grey60 module revealed two core genes (Figure 4B). Two core genes were found for the top 128 genes in the lavenderblush3 module (Figure S5B). This study found that certain core genes were more related to yield and quality. The core genes were functionally annotated by alignment with the rice database (Table 3). Further analysis of core genes and metabolites revealed that core genes were highly correlated with specific metabolites (Figure 5). We continued the correlation analysis of rice quality and metabolites (Figure S6). In addition, qRT‒PCR analysis of seven core genes was performed in this study, and the results showed that their expression patterns were basically consistent with the transcriptome data (Figure 6).

## 3. Discussion

Heterosis is a ubiquitous biological phenomenon in nature in which the F1 hybrid offspring have better performance than the parents based on selected traits, such as yield and quality. Heterosis is widely used to increase global food production, although it has complex characteristics [20]. Our team previously showed that the yield of the three-line hybrid rice variety Wufengyou T025 was significantly higher than that of its parents [1]. This study found that the YPP of two combinations of F1 hybrids, TY871 and TY398, was significantly higher than that of their parents. TY871 and TY398 showed significant heterosis in terms of yield (Table 1). EPP, SSR, and 1000-GW contributed more to TY871 yield heterosis, while EPP, PL, TGNPP, and 1000-GW contributed more to the yield heterosis of TY398. The utilization of rice heterosis has become an important method for improving rice varieties. However, it is difficult to improve the quality of hybrid rice and to simultaneously breed high-grade and high-quality rice combinations [2,21]. Hybrid F1 is a comprehensive expression of various traits from both parents, and these traits are closely related to the level of heterosis. The rice quality traits of both parents are the basis for breeding high-quality hybrid combinations. The quality of the hybrid combination was found to be better than that of the male parent because the grain type of the hybrid was longer, the grain width was narrower, and the ratio of length to width was increased, which is conducive to improving the quality of rice [9]. The parent used for this study was a high-quality variety TF, and its comprehensive rice quality traits were the best among the five varieties (Table 2). From the comprehensive data for BR, MR, HMR, CD, GC, and AC, the rice quality of the male restorer lines GH and CH was relatively poor. TY871 and TY398 integrated the rice quality traits of their male and female parents, showing certain heterosis. The results also showed that improving the CD and AC of the restorer lines can improve the quality of the hybrid rice varieties. The correlation of each sample showed the similarity of each sample. Hybrid F1 generation TY871 had a higher correlation with the parent TF (Figure 2A), and hybrid F1 generation TY398 had a higher correlation with the parent GH (Figure 2A), which was also confirmed by the rice quality data (Table 2). Metabolites are the strongest contributors to plant phenotypes and reflect the regulation of upstream genes and downstream manifestations of heterosis [22]. Changes in most metabolites were similar between the TY398 and TY871 varieties (Figure S1). The metabolites in this study were mainly organic acids and derivatives, lipids, and lipid-like molecules, and organic oxygen compounds (Figure 1). After obtaining a large amount of transcriptome and metabolome data from different species, the primary focus has converged on understanding how to mine the biological meaning in these data. Network analysis is widely used in the mining of high-throughput omics data, such as transcriptome and metabolome data [23,24,25]; for example, it could be used to discover genes and regulatory pathways related to yield and rice quality. In this study, the WGCNA method was used to identify genes related to target traits and to aggregate their classification by obtaining coexpression modules with high biological significance, which has been proven to be an efficient data mining method [26]. In this study, the honeydew1, ivory, grey60, and lavenderblush3 modules enriched by WGCNA were highly correlated with yield and rice quality and could be used as target gene modules for further research (Figure 3D). A gene coexpression network was constructed for the four modules, and it showed that the core genes *Os04g0350700* and *Os05g0154700* of the honeydew1 and grey60 modules regulate awn development, grain size, and grain number, as well as grain length and plant height. Previous studies have found that the paths significantly enriched in both hybrids were all related to yield and resistance, such as circadian rhythm (GO: 0007623), response to water deprivation (GO: 0009414), and photosynthetic genes (osa00196) [21]. In this study, via KEGG, we found significant enrichment in carbohydrate metabolism, amino acid metabolism, and energy metabolism (Figure S2B) through the comparison between the F1 generation hybrids and their parents. Furthermore, we found yield-related genes and rice quality-related metabolites (Table 3; Figure 5; Figure S6). The grain length of hybrid indica rice is closely related to rice quality, and the slender grains allow for fuller grain filling and better appearance and eating quality [27]. This study found the same phenomenon in TF varieties with slender grains (Figure 6H), and the rice quality was rated as national standard high-quality rice grade one. TY871 and TY398 (Figure 6H) were rated as national standard high-quality rice grade two. Previous studies have shown that grain metabolites may be one of the central factors affecting the nutritional quality and eating quality of rice [17,18,19]. In this study, NEG_M341T662 metabolites were found to be highly correlated with rice quality (Figure S6), and NEG_M341T662 was highly connected to the core genes *Os06g0254300* and *Os03g0168100* (Figure 5). Whether the metabolite NEG_M341T662 affects rice quality deserves further investigation. This study offers a foundation for analyzing the high yield and high quality of three-line hybrid rice.

## 4. Materials and Methods

### 4.1. Plant Materials, Field Experiments, and Sampling

In this study, we planted the hybrid Taiyou 871 (TY871) and its parental lines Changhui 871 (CH) and Taifeng B (TF), and the hybrid Taiyou 398 (TY398) and its parental lines Guanghui 398 (GH) and Taifeng B (TF), in the experimental field of Jiangxi Agricultural University. The field cultivation and collection of the experimental materials fully complied with relevant institutional, national, and international guidelines and legislation. The experimental materials were divided into three stages of seeding (sowing dates seven days apart), including three replicates of all treatments, in autumn 2020. The glumes of each variety were marked on the day of rice flowering, and endosperm tissue of each variety was collected on the 9th day after flowering. Three biological replicates were sampled for each variety for RNA extraction, and six biological replicates were sampled per variety for metabolite extraction. All samples were flash frozen in liquid N and subsequently stored at −80 °C for RNA and metabolite extraction.

### 4.2. RNA Extraction and Profiling Analysis

RNA was extracted, and library construction was performed according to Xiong et al. [28] and sequenced using an Illumina HiSeqTM 2500 device by Gene Denovo Biotechnology Co. (Guangzhou, China). RNA differential expression analysis between two different groups was performed by DESeq2 [29] software between two different groups. Genes/transcripts with a false discovery rate below 0.05 and absolute fold change ≥ 2 were considered differentially expressed genes/transcripts. Kyoto Encyclopedia of Genes and Genomes (KEGG) enrichment analysis and Gene Ontology (GO) enrichment were conducted based on the DEGs of each pairwise comparison (False discovery rate < 0.05). Bioinformatic analysis was conducted using Omicsmart, a real-time interactive online platform for data analysis (http://www.omicsmart.com, accessed on 18 July 2021). The R package software and weighted gene coexpression network analysis (WGCNA) were used to analyze differentially expressed genes (DEGs) among different samples, to divide the modules of the yield and quality data and to screen the core genes [30,31]. To identify biologically significant modules, module eigengenes were used to calculate the correlation coefficient with samples or sample traits. The intramodular connectivity (K.in) and module correlation degree (MM) of each gene were calculated by the R package for WGCNA, and genes with high connectivity tended to be hub genes that might have important functions. Correlation analysis was performed using module eigengenes with data for specific traits or phenotypes. Pearson correlations between each gene and trait data under the module were also calculated for the most relevant module (positive correlation and negative correlation) corresponding to each phenotype data, and the gene significance value (GS) was obtained. The networks were visualized using Cytoscape (v3.3.0) software [32].

### 4.3. Metabolite Extraction, Identification, and Profiling Analysis

The metabolites were extracted according to Dunn et al. [33] and Sangster et al. [34]. Chromatographic separation was accomplished in a Thermo Ultimate 3000 (Thermo Fisher Scientific, Shanghai, China) system equipped with an ACQUITY UPLC^®^ HSS T3 column (150 × 2.1 mm, 1.8 µm, Waters) maintained at 40 °C. The temperature of the autosampler was 8 °C. Gradient elution of analytes was carried out with 0.1% formic acid in water (C) and 0.1% formic acid in acetonitrile (D) or 5 mM ammonium formate in water (A) and acetonitrile (B) at a flow rate of 0.25 mL/min. Injection of 2 μL of each sample was performed after equilibration. An increasing linear gradient of solvent B (*v*/*v*) was used as follows: 0–1 min, 2% B/D; 1–9 min, 2–50% B/D; 9–12 min, 50–98% B/D; 12–13.5 min, 98% B/D; 13.5–14 min, 2–98% B/D; and 14–20 min, 2% D-positive model (14–17 min, 2% B-negative model). The ESI-MSn experiments were executed on a Thermo Q Exactive mass spectrometer (Thermo Fisher Scientific, Shanghai, China) with spray voltages of 3.8 kV and −2.5 kV in positive and negative modes, respectively. Sheath gas and auxiliary gas were set at 30 and 10 arbitrary units, respectively. The capillary temperature was 325 °C. The analyzer scanned over a mass range of *m*/*z* 81–1000 for full scan at a mass resolution of 70,000. Data-dependent acquisition (DDA) MS/MS experiments were performed with an HCD scan. The normalized collision energy was 30 eV. Dynamic exclusion was implemented to remove some unnecessary information in the MS/MS spectra. The format of the raw data files was converted into mzXMLformat using Proteowizard (v3.0.8789). The R package (v3.3.2) XCMS [35] was used to perform peak identification, peak filtration, and peak alignment for each metabolite. The main parameters were set as follows: bw = 5, ppm = 15, peakwidth = c(5,30), mzwid = 0.01, mzdiff = 0.01, and method = “centWave”. The mass-to-charge ratio (*m*/*z*), retention time and intensity, and positive and negative precursor molecules were used for subsequent analysis. Peak intensities were batch normalized to the total spectral intensity. Identification of metabolites was based on the exact molecular formula (molecular formula error < 20 ppm). Peaks were matched with Metlin (http://metlin.scripps.edu, accessed on 18 July 2021) and MoNA (https://mona.fiehnlab.ucdavis.edu/, accessed on 18 July 2021) to confirm annotations for metabolites.

As a preliminary visualization of differences between various groups of samples, the unsupervised dimensionality reduction method principal component analysis (PCA) was applied to all samples using R package models (http://www.r-project.org/, accessed on 18 July 2021). The Pearson correlation coefficient and *p*-value were calculated by the R functions, cor. and cor.test. The closer the correlation coefficient is to 1, the higher the similarity of metabolic abundance. Correlations were considered significant when *p* ≤ 0.05. A correlation heatmap was drawn by the R corrplot package [36]. The abundances of differential metabolites (DMs) were normalized by z score and hierarchically clustered by the R package pheatmap [37] to show accumulation differences between the two groups. Metabolites were mapped to KEGG metabolic pathways for annotation and enrichment analysis [38].

### 4.4. Yield and Rice Quality Determination

Five rice plants of each variety were sampled after the rice matured to investigate panicle length (PL), total grain number per panicle (TGNPP), empty grains per panicle, 1000-grain weight (1000-GW), effective panicle per plant (EPP), primary branch number (PBN), secondary branch number (SBN), and yield per plant (YPP), and the rice seed setting rate (SSR) was calculated. After the rice matured, three biological replicates were taken from each variety, and the full grains were picked out by an NP-4350 type wind separator. After 3 months of indoor storage, the brown rice rate (BR), milled rice rate (MR), head milled rice rate (HMR), chalkiness degree (CD), gel consistency (GC), and amylose content (AC) of the rice were determined based on standard GB/T 17891-2017 [39].

### 4.5. Quantitative Real-Time Polymerase Chain Reaction (qRT‒PCR)

Total RNA was isolated from rice grain endosperm using a Takara Plant MiniBEST RNA Extraction Kit (Takara, Nanchang, China), and cDNA was synthesized using PrimeScriptTM II reverse transcriptase (Takara, Nanchang, China) according to the manufacturer’s instructions. qRT‒PCR was conducted using a 7500 Real-Time PCR System (Applied Biosystems, Shanghai, China) in conjunction with a SYBR Green PCR Kit (Takara, Nanchang, China) with a reaction volume of 20 μL [28]. The *OsActin* gene was used as a control. The sequences of the qRT‒PCR primers are shown in Table S12.

### 4.6. Statistical Analysis

WPS 2021 software v13.18.0 was used to sort the yield and quality data, and calculate the average value. SPSS software v18.0 was used to perform variance analysis on the yield and quality data.

## 5. Conclusions

In this study, we constructed a coexpression network of weighted genes related to yield and rice quality and identified nine gene closely related modules. Four gene modules with the strongest correlation to the target trait were selected for in-depth analysis. One core gene was found in the honeydew1 module, two core genes were found in the ivory module, two core genes were found in the grey60 module, and two core genes were found in the lavenderblush3 module. Through functional annotation, we found that the core genes *Os04g0350700* and *Os05g0154700* from the honeydew1 and grey60 modules contained functions regulating awn development, grain size, and grain number and regulating grain length and plant height, respectively. Among them, the metabolite NEG_M341T662 is highly connected to the core genes *Os06g0254300* and *Os03g0168100*. The research results provide theoretical support for analyzing the breeding direction of three-line hybrid rice with high yield and high quality through the utilization of heterosis.

## Figures and Tables

**Table 1:** Differences in yield and yield components of two hybrid rice varieties (*Oryza sativa* L.) and their parents.

Variety	EPP	PL (cm)	PBN	SBN	TGNPP	SSR (%)	1000-GW (g)	YPP (g)
TY398	12.67 ± 0.58 a	25.63 ± 0.46 a	13.78 ± 0.69 bc	43.78 ± 5.23 ab	196.83 ± 4.01 b	81.79 ± 0.85 a	23.55 ± 0.59 b	39.25 ± 4.65 ab
GH	9.67 ± 0.58 b	22.68 ± 0.19 b	12.47 ± 1.12 c	36.58 ± 6.57 b	163.33 ± 8.97 c	80.48 ± 1.28 ab	20.53 ± 0.32 cd	22.94 ± 1.52 c
TF	8.00 ± 1.00 b	21.81 ± 0.59 b	12.00 ± 0.58 c	23.22 ± 2.12 c	109.89 ± 0.69 d	70.65 ± 1.16 d	19.95 ± 0.18 e	13.14 ± 0.88 d
TY871	12.67 ± 0.58 a	27.38 ± 2.00 a	15.11 ± 0.69 b	52.22 ± 1.71 a	215.00 ± 2.52 b	78.55 ± 0.65 b	24.97 ± 0.09 a	44.91 ± 2.43 a
CH	9.00 ± 1.00 b	27.24 ± 0.17 a	18.00 ± 0.00 a	51.00 ± 3.18 a	253.44 ± 14.69 a	75.61 ± 0.97 c	20.95 ± 0.42 c	35.96 ± 1.99 b

TY871: Taiyou 871; CH: Changhui 871; TF: Taifeng B; TY398: Taiyou 398; GH: Guanghui 398; PL: panicle length; TGNPP: total grain number per panicle; SSR: seed setting rate; 1000-GW: 1000-grain weight; EPP: effective panicle per plant; PBN: primary branch number; SBN: secondary branch number; and YPP: yield per plant. Different lowercase letters indicate significant differences at *p* < 0.05. The same applies below. The data are shown as the mean ± s.e.m. (*n* = 5).

**Table 2:** Differences in rice quality of two hybrid rice varieties (*Oryza sativa* L.) and their parents.

Variety	BR	MR	HMR	CD	GC	AC
TY398	75.39 ± 0.07 b	63.23 ± 0.25 d	49.73 ± 2.99 bc	3.67 ± 0.34 bc	73.00 ± 1.00 a	16.03 ± 0.81 ab
GH	77.18 ± 0.36 a	67.25 ± 0.10 a	53.72 ± 0.74 ab	4.39 ± 0.13 ab	63.33 ± 3.79 b	17.15 ± 0.58 a
TF	73.71 ± 0.26 c	64.15 ± 0.23 c	55.08 ± 2.06 b	2.08 ± 0.48 d	80.00 ± 1.00 a	13.93 ± 0.07 c
TY871	74.03 ± 0.66 c	65.29 ± 0.30 b	56.85 ± 2.41 a	3.39 ± 0.37 c	64.33 ± 2.52 b	15.14 ± 0.84 bc
CH	72.54 ± 0.25 d	61.82 ± 0.09 e	47.31 ± 0.28 d	4.80 ± 0.17 a	49.00 ± 4.36 c	16.23 ± 0.34 ab

TY871: Taiyou 871; CH: Changhui 871; TF: Taifeng B; TY398: Taiyou 398; GH: Guanghui 398; BR: brown rice rate; MR: milled rice rate; HMR: head milled rice rate; CD: chalkiness degree; GC: gel consistency; and AC: amylose content. Different lowercase letters indicate significant differences at *p* < 0.05. The data are shown as the mean ± s.e.m. (*n* = 3).

**Table 3:** Functional annotation of core genes in yield- and quality-related modules.

Module	Core Gene	Gene Function
Honeydew1	*Os04g0350700*	Basic helix–loop–helix protein, Regulation of awn development, grain size, and grain number
Ivory	*Os06g0318500*	Sodium/hydrogen exchanger
Ivory	*Os03g0793700*	RmlC-like jelly roll fold domain containing protein
Grey60	*Os05g0363200*	UDP-glucuronic acid decarboxylase
Grey60	*Os05g0154700*	Kinesin 13 protein, Regulation of grain length and plant height
Lavenderblush3	*Os06g0254300*	EF-hand calcium binding protein
Lavenderblush3	*Os03g0168100*	Late embryogenesis abundant protein repeat containing protein

## Data Availability

The RNA-seq raw sequencing data from this study have been deposited in the Genome Sequence Archive in BIG Data Center (http://bigd.big.ac.cn/, accessed on 20 July 2021), under the accession number: CRA009572.

## Supplementary Materials

The following supporting information can be downloaded at: https://www.mdpi.com/article/10.3390/ijms232112934/s1.

## Abbreviations

WGCNA	weighted gene coexpression network
GO	Gene Ontology
KEGG	Kyoto Encyclopedia of Genes and Genomes
DEG	differentially expressed gene
PCA	principal component analysis
DMs	differential metabolites
TY871	Taiyou 871
CH	Changhui 871
TF	Taifeng B
TY398	Taiyou 398
GH	Guanghui 398
PL	panicle length
TGNPP	total grain number per panicle
1000-GW	1000-grain weight
EPP	effective panicle per plant
PBN	primary branch number
SBN	secondary branch number
YPP	yield per plant
SSR	seed setting rate
BR	brown rice rate
MR	milled rice rate
HMR	head milled rice rate
CD	chalkiness degree
GC	gel consistency
AC	amylose content
qRT‒PCR	quantitative real-time polymerase chain reaction
