# Supplementary material for: Transcriptome and Metabolome Analysis Provides Insights into the Heterosis of Yield and Quality Traits in Two Hybrid Rice Varieties (Oryza sativa L.)
Source: Int J Mol Sci. 2022 Oct 26;23(21):12934. doi: 10.3390/ijms232112934 (PMC9654843; doi:10.3390/ijms232112934)
Supplement: Supplementary file 1 [file ijms-23-12934-s001.zip › Figure S1.pdf]

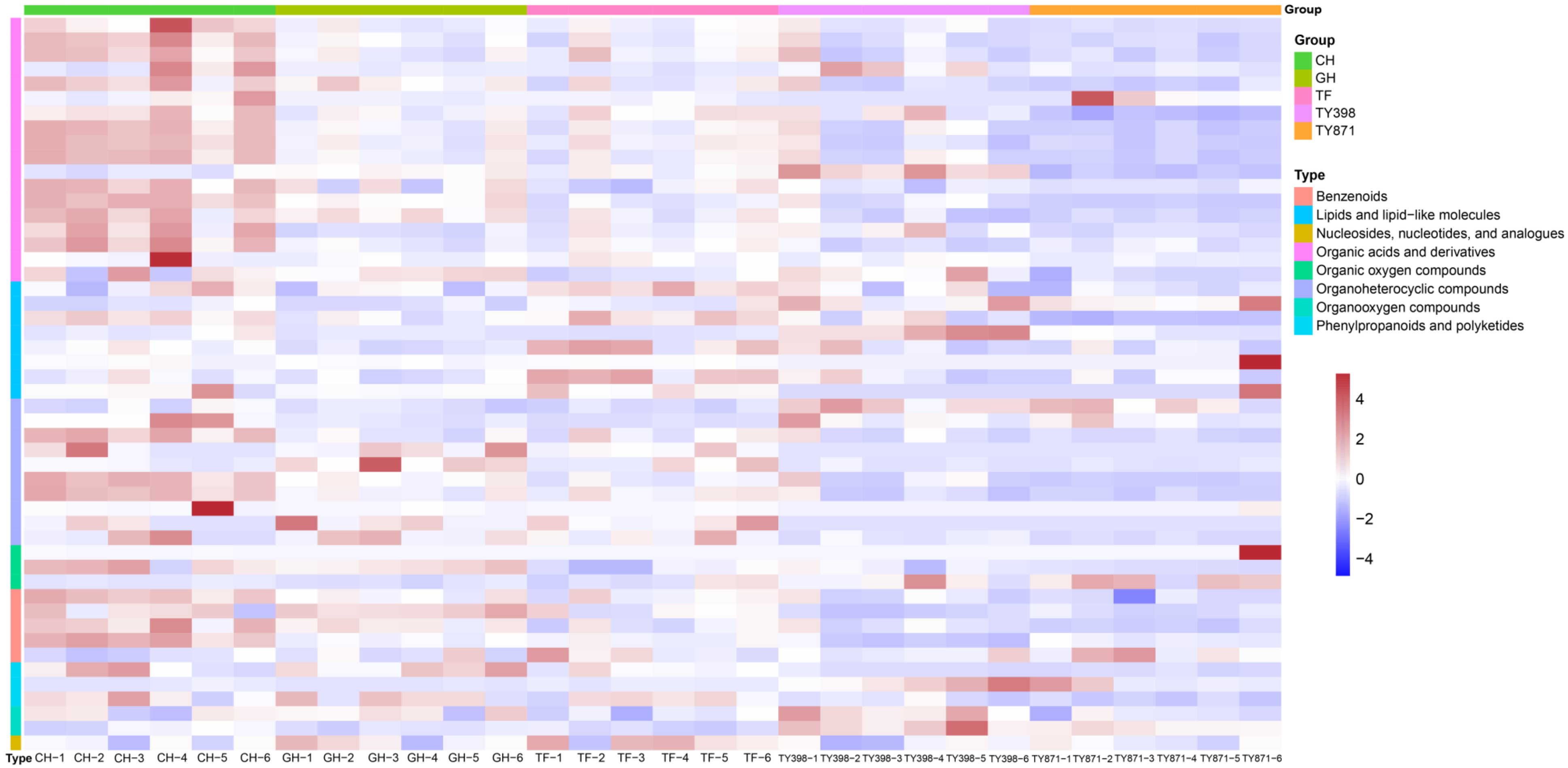

Figure S1. Heatmap presentation of the variation in the metabolites with different rice varieties. Each line in this heatmap represents a specific metabolite. The value of each compound indicates the relative content that was normalized directly on a similar graphical scale and is scaled by median values for each compound. Red indicates high content, and blue indicates low content of metabolites in the sample.
