# Supplementary material for: Transcriptome and Metabolome Analysis Provides Insights into the Heterosis of Yield and Quality Traits in Two Hybrid Rice Varieties (Oryza sativa L.)
Source: Int J Mol Sci. 2022 Oct 26;23(21):12934. doi: 10.3390/ijms232112934 (PMC9654843; doi:10.3390/ijms232112934)
Supplement: Supplementary file 1 [file ijms-23-12934-s001.zip › Figure S2.pdf]

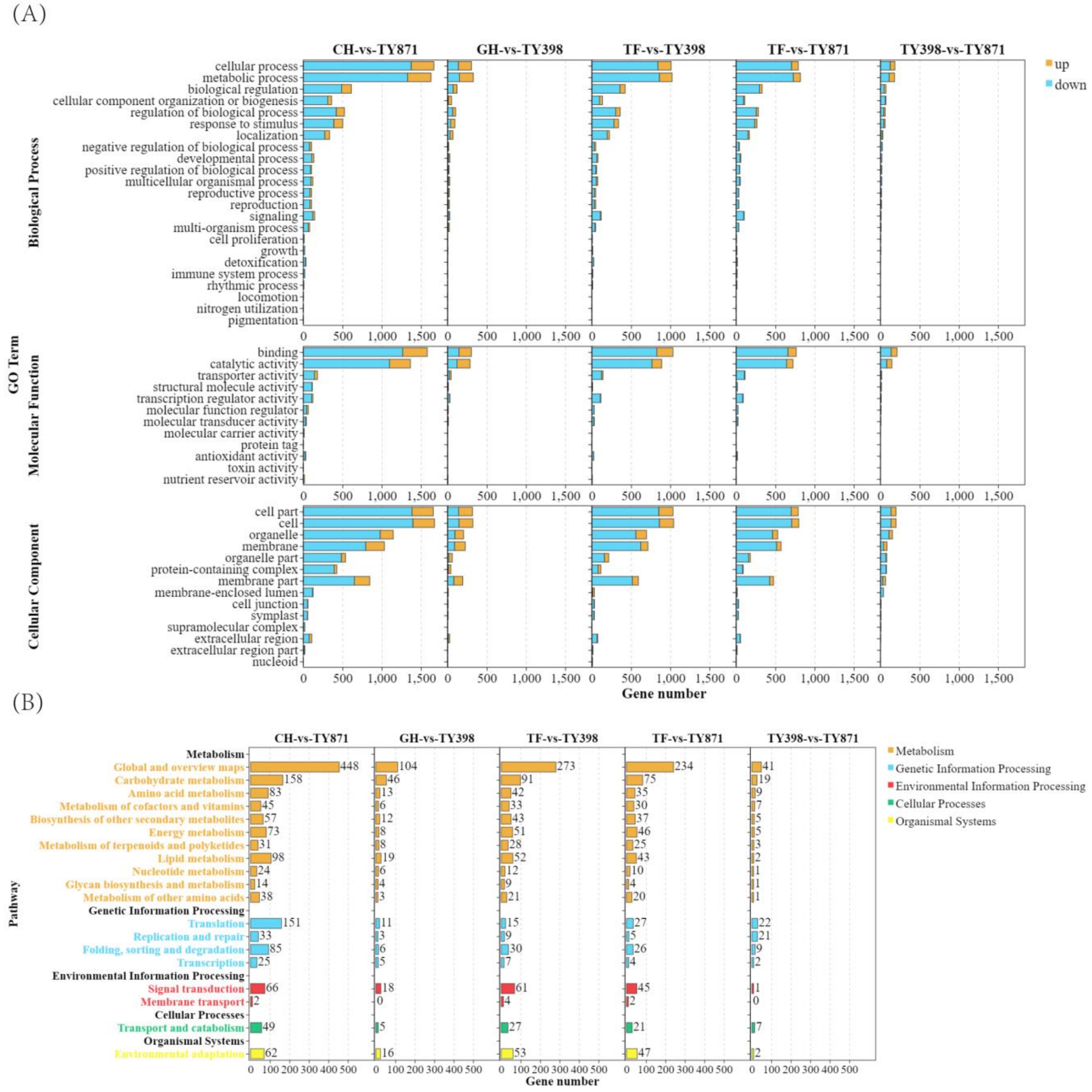

Figure S2. GO and pathway analysis. (A) GO term. The horizontal axis is the number of genes, and the vertical axis is the GO term. (B) Pathway. The horizontal axis is the number of genes, and the vertical axis is the pathway.
