# Supplementary material for: Transcriptome and Metabolome Analysis Provides Insights into the Heterosis of Yield and Quality Traits in Two Hybrid Rice Varieties (Oryza sativa L.)
Source: Int J Mol Sci. 2022 Oct 26;23(21):12934. doi: 10.3390/ijms232112934 (PMC9654843; doi:10.3390/ijms232112934)
Supplement: Supplementary file 1 [file ijms-23-12934-s001.zip › Figure S3.pdf]

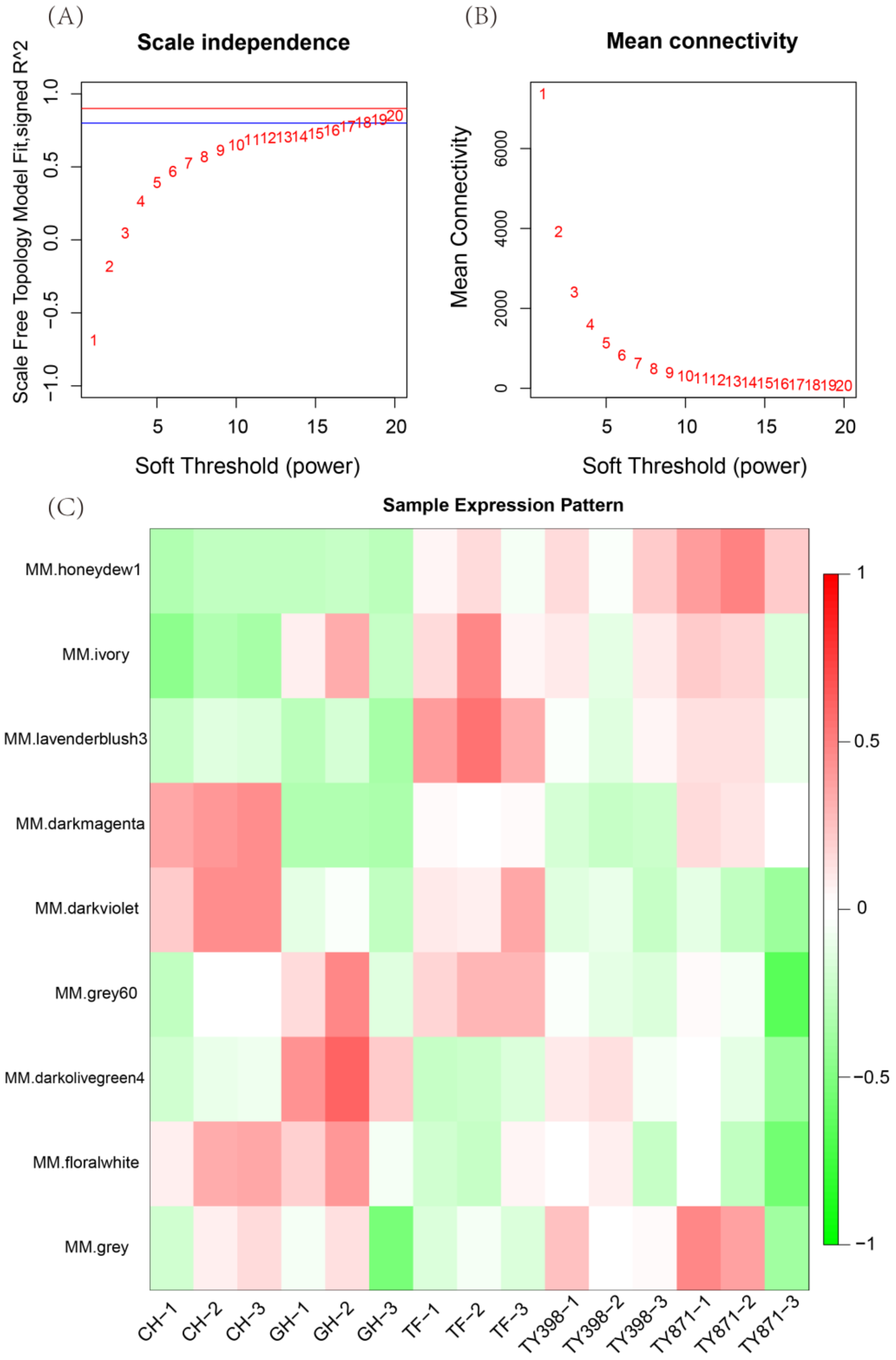

Figure S3. Power value graph and sample expression pattern analysis. (A) The horizontal axis represents the power value, the vertical axis represents the correlation coefficient, the blue horizontal line represents the correlation coefficient of 0.8, and the red horizontal line represents the correlation coefficient of 0.9. (B) The abscissa represents the power value, and the ordinate represents the average connectivity of genes. (C) The abscissa is the sample, the ordinate is the module, and the eigenvalues of the module are used for plotting. Red represents high expression, and green represents low expression.
