# Supplementary material for: Transcriptome and Metabolome Analysis Provides Insights into the Heterosis of Yield and Quality Traits in Two Hybrid Rice Varieties (Oryza sativa L.)
Source: Int J Mol Sci. 2022 Oct 26;23(21):12934. doi: 10.3390/ijms232112934 (PMC9654843; doi:10.3390/ijms232112934)
Supplement: Supplementary file 1 [file ijms-23-12934-s001.zip › Figure S4.pdf]

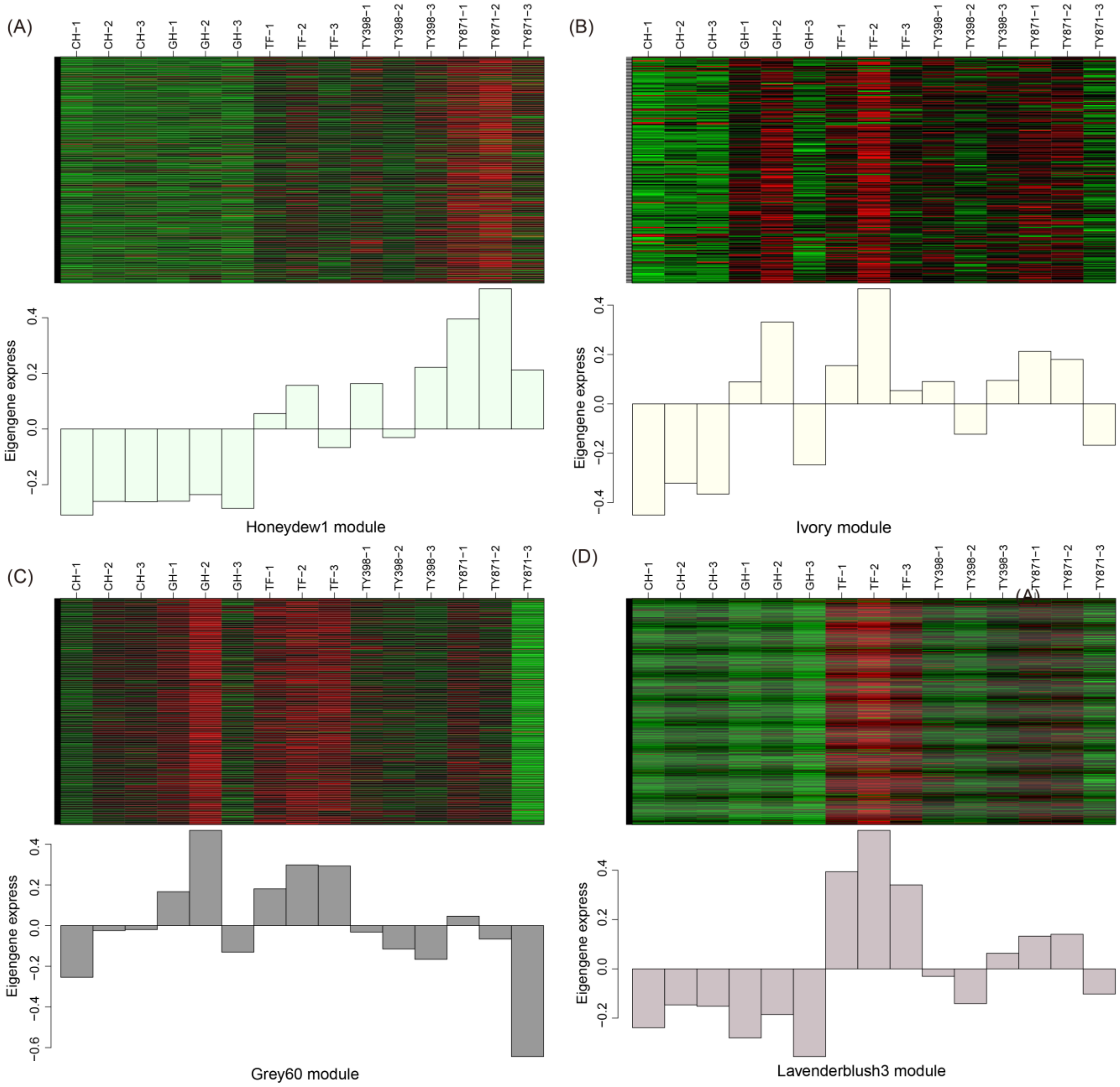

Figure S4. Heatmap of the gene expression patterns of each module. (A) Honeydew1 module; (B) Ivory module; (C) Grey60 module; (D) Lavenderblush3 module.
