# Supplementary material for: Transcriptome and Metabolome Analysis Provides Insights into the Heterosis of Yield and Quality Traits in Two Hybrid Rice Varieties (Oryza sativa L.)
Source: Int J Mol Sci. 2022 Oct 26;23(21):12934. doi: 10.3390/ijms232112934 (PMC9654843; doi:10.3390/ijms232112934)
Supplement: Supplementary file 1 [file ijms-23-12934-s001.zip › Table S12.pdf]

Table S12 Sequences of the qRT-PCR primers used.

Gene ID	Forward primer	Amplicon length (bp)	Reverse primer	Amplicon length (bp)
Os04g0350700	CCTTCCAAAGATATGAGCCATAT GCC	26	GTGGCTCCCATTTGCATAGCG	21
Os06g0318500	GCACTCCACTGATAGGGAAGTAG C	24	GCAAATATGTGCCTGGTTGTGATTC	25
Os03g0793700	AGGTGTTCGAGAGGCAGCG	19	CGGTGAGGCTGGACGGCT	18
Os05g0363200	ATGGACTATCACCGTGGTGCC	21	CTCCTGGTCTGCTTGCCATC	20
Os05g0154700	AGCAAAAGCTGATGGCACC	19	GCTGAGAGAGCGGAGGAGC	19
Os06g0254300	GACCATTGAAGGGATTGTTGCAA TCG	26	GTCACTCCCGTGCATTCC	18
Os03g0168100	CCGCCTCCGAGGCAGGGAG	19	GTCTTGTGCTTGGCCTGCTCCG	22
OsActin	CTTCATAGGAATGGAAGCTGCGG GTA	26	CGACCACCTTGATCTTCATGCTGCTA	26
